# Lessons on the Anatomy, Physiology and Hygiene of Infancy and Childhood

**Published:** 1900-12

**Authors:** 


LESSONS ON THE ANATOMY, PHYSIOLOGY AND HYGIENE OF INFANCY AND CHILDHOOD. Consisting of extracts from lectures given at Rush Medical College, by ALFRED C. COTTON, A. M., M. D., Professor of Diseases of Children; Accoucheur and Physician for Diseases of Children, Presbyterian Hospital. 100 illustrations. Chicago: Chicago Medical Book Co. [Price, cloth, $1.50 net.]

The author of this book has been a teacher of medicine for twenty years. Finding that the greatest drawback for a thorough comprehension of the diseases of childhood on the part of his senior students, was their ignorance of the special anatomy, physiology and hygiene of early life, he began his course in pedriatrics by a series of lectures to the junior class on these subjects. The book embodies these lectures. Teachers of pediatrics and students interested in this branch of medicine will both welcome the attempt which Dr. Cotton has made to place before the student knowledge of which he is in confessed need and which he can hardly get together for himself.

The book is illustrated by many plates and engravings, for the most part new. The chapters on anatomy present in clear brief manner the deviations from the adult type which obtain in early life. Then follow chapters on growth in which the changes that occur as age advances are enumerated. The greater part of the book deals with the subject of hygiene, a knowledge which is often painfully lacking in the young practitioner.

While Dr. Cotton's book is good it is disappointing, in that with careful editing it might have been so much better. A second edition will, we trust, remedy certain too obvious defects.—M. J. F.

